# HPV vaccination intention and its association with vaccine hesitancy and health beliefs among unvaccinated Chinese medical college students: A cross-sectional study

**DOI:** 10.1371/journal.pone.0354119

**Published:** 2026-07-20

**Authors:** Ying-Ai Cui, Yuan Li, Liping Yang, Zheng-Ai Cui

**Affiliations:** 1 School of Nursing, Guangdong Medical University, Dongguan, Guangdong, China; 2 Finance Department, Guangdong Medical University, Dongguan, Guangdong, China; 3 International Medical and Special Needs Service Center, The First Dongguan Affiliated Hospital of Guangdong Medical University, Dongguan, Guangdong, China; 4 School of Humanities and Management, Guangdong Medical University, Dongguan, Guangdong, China; Nazarbayev University School of Medicine, KAZAKHSTAN

## Abstract

**Background:**

Human papillomavirus (HPV) infection poses a significant public health challenge, and HPV vaccination uptake among Chinese college students remains suboptimal. Therefore, it is essential to conduct ongoing, in-depth investigations into the psychological and cognitive mechanisms of HPV vaccination intention. This study aims to explore the associations among HPV vaccination intention, vaccine hesitancy, and health beliefs among Chinese medical college students.

**Methods:**

A cross-sectional survey was conducted from June 16 to July 16, 2024, to assess sociodemographic characteristics, vaccine hesitancy, and health belief model (HBM) among college students at Guangdong Medical University. Descriptive statistics, univariate analysis, and binary logistic regression were performed to identify factors associated with HPV vaccination intentions among unvaccinated students.

**Results:**

Among 3,584 unvaccinated participants, 73.16% expressed willingness to receive the HPV vaccine. The key significant associations identified included gender (male) (OR: 0.26; 95% CI: 0.22 ~ 0.31; *p*  <  0.001), parental occupation (employees in enterprises, commercial, and service industries (including self-employed)) (OR: 1.43; 95% CI: 1.18 ~ 1.75; *p*  <  0.001), and information sources (hospitals or doctors) (OR: 1.37; 95% CI: 1.15 ~ 1.64; *p*  =  0.001). Vaccination intention was also directly associated with the perceived necessity (OR: 0.91; 95% CI: 0.88 ~ 0.94; *p*  <  0.001) and importance (OR: 1.07; 95% CI: 1.03 ~ 1.10; *p*  <  0.001) of vaccine hesitancy, as well as the perceived benefits of behavior for the HBM (OR: 1.21; 95% CI: 1.12 ~ 1.32; *p* <  0.001).

**Conclusions:**

In this sample, Chinese medical college students demonstrated a relatively high willingness to receive HPV vaccination. Willingness was positively associated with perceived necessity, importance, and benefits of vaccination. Integrating structured clinician-delivered counseling into curricula, using parental occupation data for targeted subsidies, and embedding classroom sessions comparing personal HPV risk with vaccine safety data may improve uptake. Government evaluation of gender-neutral vaccination policies and provision of male-inclusive campus services are also recommended.

## Introduction

Human papillomavirus (HPV) infection poses a major global public health challenge. Despite the high preventability of HPV infection, the situation in China remains severe [1,2]. College students, who are in the early stage of sexual activity, are highly susceptible to HPV and constitute a key population for HPV prevention and control [3]. However, the current status of HPV vaccination among Chinese college students is not encouraging. Data have indicated that the peak first-dose HPV vaccination rate among Chinese youth aged 20–24 years is only 14.02% [4]; among female college students in northern China, despite a slightly higher vaccination rate of 18.11%, the willingness to receive the vaccine remains low, with a notable 41.71% explicitly stating their unwillingness to be vaccinated [5]. Additionally, vaccination behavior significantly differs across genders, geographical locations, and regions [5]. Consequently, it is imperative to conduct an ongoing, in-depth exploration of the psychological and cognitive mechanisms underlying the decision-making process regarding HPV vaccination among Chinese college students.

For a long time, the health belief model (HBM) has served as a classic theoretical framework for elucidating vaccination behavior. According to this model, individuals’ preventive health decisions are predominantly shaped by factors such as perceived benefits of preventive actions, perceived susceptibility to diseases, and perceived severity of disease [6]. However, the HBM, which is primarily grounded in a “rational choice” perspective, may fail to fully capture the complex social and psychological factors underlying contemporary vaccine hesitancy [7], which has been recognized as a major barrier to vaccination [8]. To obtain a more comprehensive understanding of this phenomenon, the World Health Organization’s Strategic Advisory Group of Experts on Immunization (SAGE) proposed the “3C” (complacency, convenience, and confidence) model as a metric for assessing vaccine hesitancy [9]. This model offers a complementary perspective for analyzing vaccination behaviors and demonstrates high applicability within the Chinese context. [10,11]. Within China’s unique policy context, college students are required to fully self-fund their HPV vaccinations, as these are not incorporated into the national immunization program [12]. The cost per dose varies from 360 CNY to 1331 CNY, depending on the vaccine type. This real-world situation introduces both structural and psychological barriers, which are frequently challenging to comprehensively elucidate within a singular theoretical construct.

Notably, although the aforementioned two theoretical frameworks play a pivotal role in predicting the willingness to receive vaccination, there remains a dearth of empirical evidence integrating them for application in studies on HPV vaccination among Chinese college students. Current studies predominantly concentrate on a singular dimension or isolated theoretical perspective, resulting in inconsistent findings and limited explanatory capacity. Such a fragmented tendency further complicates the elucidation of how health beliefs and vaccine hesitancy collectively influence vaccination decisions, thereby constraining the identification of precise intervention targets.

To address this issue, this study employs a cross-sectional design to investigate the associations among willingness to receive HPV vaccination, vaccine hesitancy, and health beliefs among students at a Chinese medical university. By simultaneously examining variables from both the HBM and the vaccine hesitancy framework. This study aims to delineate their independent associations with HPV vaccination intention in this population. While it does not formally test the structural integration of these two frameworks, the findings offer a preliminary empirical basis that may inform the development of more integrated theoretical models in future research. Furthermore, the study provides evidence to guide targeted campus-based immunization strategies within medical university settings.

## Materials and methods

### Survey design and data collection

This cross-sectional study was carried out at Guangdong Medical University between June 16 and July 16, 2024. A convenience sampling approach was employed, and the data were collected through an online questionnaire hosted on the Chinese survey platform “Wenjuanxing” (www.wjx.cn). The survey link was shared with full-time students via WeChat.

A total of 5,384 individuals participated in the survey. To ensure data quality, 456 invalid responses were excluded based on three pre-defined criteria: (1) completion time < 180 seconds (n = 199), given that the 35-item survey (including single-choice, multiple-choice, and fill-in-the-blank questions) allowed an average of approximately 5 seconds per item—insufficient for attentive reading and responding, especially for text-entry items; (2) inconsistent or implausible responses (n = 45), operationalized as a self-reported age below 14 or above 30, which are extreme outliers relative to the typical full-time student age range (16–25 years) as verified by the university’s student affairs office; (3) unverifiable or inaccurate academic/professional information (n = 212). After exclusion, this yielded 4,928 valid questionnaires (validity rate: 91.53%). Among the participants with valid responses, 3,584 participants (unvaccinated rate: 72.73%) had not received the HPV vaccine or were in the process of scheduling an appointment for it. Therefore, the final analysis included 3,584 Chinese college students.

### Ethical considerations

Ethical approval for this investigation was granted by the Clinical Research Ethics Committee of the Affiliated Hospital of Guangdong Medical University (approval NO. PJKT2023−126). An online informed consent document was reviewed and signed by each participant before enrollment. Furthermore, all prospective participants were assured of the voluntary nature of the study and their prerogative to withdraw their consent at any given moment.

### Variables and instruments

The survey included sociodemographic characteristics, the vaccine hesitancy scale, and the health belief model.

### Sociodemographic characteristics

The following characteristics were assessed in this survey: gender, age, monthly consumption, household income, main economic source, parents’ occupations, sources/channels of information about HPV, and hearing negative news about the HPV vaccine and HPV vaccination intention. HPV vaccination intention was assessed using a single binary item: “If you had the opportunity to receive the HPV vaccine, would you be willing to be vaccinated?” Response options were “Yes” or “No” (Table 1).

**Table 1:** Sociodemographic characteristics of the participants (n = 3584).

Variable	Term	N (%)
Gender	Female	1996(55.69)
	Male	1588(44.31)
Age (M ± SD)		20.63 ± 1.74
Average Monthly Consumption Level (CNY)	1000-1500	1839(51.31)
	1501-2000	987(27.54)
	2001-3000	326(9.10)
	>3000	110(3.07)
Average Monthly Household Income(CNY)	<1500	96(2.68)
	1500-2500	224(6.25)
	2501-4000	581(16.21)
	4001-8000	1265(35.30)
	>8000	1418(39.56)
Main Economic Source	Parents	3440(95.98)
	Other family members, Relatives, Friends	569(15.88)
	Government fundings	182(5.08)
	Scholarships	597(16.66)
	Part-time job while studying	1158(32.31)
	Self-employment	68(1.90)
	Student loans	225(6.28)
	Others	238(6.64)
Parents’ Occupations	Personnel of state organs, Party-mass organizations and public institutions	252(7.03)
	Employees in enterprises, commercial, and service industries (including self-employed)	1001(27.93)
	Workers	969(27.04)
	Farmers	645(18.00)
	Teachers and medical professionals	295(8.23)
	Others (e.g., military personnel, freelancers)	1204(33.59)
	Unemployed	241(6.72)
Sources/channels of information about HPV	Schools/teachers/classmates	2920(84.15)
	Hospital/Doctor	1885(54.32)
	Family/relatives/friends	1168(33.66)
	Social media (WeChat, Weibo, QQ, etc.)	2702(77.87)
	Internet (website, email, newsletter, etc.)	2620(75.50)
	TV/radio/newspapers/magazines	1327(38.24)
	Advertising	797(22.97)
	Government	705(20.32)
	Community Activities	727(20.95)
	Others	281(8.10)
Heard the negative news about the HPV vaccine?	No	1811(50.53)
	Yes	1773(49.47)
HPV Vaccination Intention?	No	962(26.84)
	Yes	2622(73.16)

Continuous variables are summarized as the mean (M) ± standard deviation (SD), whereas categorical variables are reported as counts (N) and proportions (%), same below.

### Vaccine hesitancy

The vaccine hesitancy scale utilized in this study was developed by Wei et al. [11] (Table 2). This scale includes 12 items across 3 dimensions: necessity (consisting of 4 items), importance (consisting of 5 items), and safety (consisting of 3 items). Each item was scored on a 5-point Likert scale, where a score of 1 indicated “strongly disagree” and a score of 5 indicated “strongly agree”. Notably, the items assessing the necessity and safety dimensions were reverse-coded. The overall Cronbach’s α value for this scale was 0.732. And, the Cronbach’s α values for the necessity, importance, and safety dimensions of the scale were 0.922, 0.888, and 0.848, respectively.

**Table 2:** Descriptive statistics of HPV vaccine hesitancy.

Term	Strongly disagree	Disagree	Neutral/Unsure	Agree	Strongly agree	Score
	N (%)	N (%)	N (%)	N (%)	N (%)	(M ± SD)
1. Current treatments are already very good, and HPV vaccination is not that necessary.	1238(34.54)	1678(46.82)	437(12.19)	167(4.66)	64(1.79)	1.92 ± 0.90
2. HPV infection can be cleared up by one’s own immunity, so there is no need for vaccination.	1523(42.49)	1567(43.72)	323(9.01)	129(3.60)	42(1.17)	1.77 ± 0.84
3. HPV vaccination is not mandatory by the government, so it is not necessary.	1279(35.69)	1649(46.01)	456(12.72)	154(4.30)	46(1.28)	1.90 ± 0.87
4. I am in good health and do not need HPV vaccination.	1259(35.13)	1551(43.28)	546(15.23)	171(4.77)	57(1.59)	1.94 ± 0.91
***Necessity***						7.54 ± 3.18
5. HPV vaccination is the best way to protect me (or my girlfriend or future wife) from cervical cancer.	43(1.20)	131(3.66)	389(10.85)	1863(51.98)	1158(32.31)	4.11 ± 0.82
6. HPV vaccination protects my family (husband/wife, children) from infection.	65(1.81)	275(7.67)	608(16.96)	1699(47.41)	937(26.14)	3.88 ± 0.94
7. HPV vaccination is effective in preventing cervical cancer.	23(0.64)	43(1.20)	317(8.84)	1984(55.36)	1217(33.96)	4.21 ± 0.70
8. HPV vaccination is very important for my health.	27(0.75)	49(1.37)	428(11.94)	1877(52.37)	1203(33.57)	4.17 ± 0.74
9. HPV vaccination is effective in preventing some other diseases (e.g., warts).	26(0.73)	37(1.03)	368(10.27)	1948(54.35)	1205(33.62)	4.19 ± 0.71
***Importance***						20.56 ± 3.28
10. I am worried about the side effects of HPV vaccination.	78(2.18)	354(9.88)	1184(33.04)	1615(45.06)	353(9.85)	3.51 ± 0.88
11. I am worried that the HPV vaccine I receive is counterfeit or expired.	92(2.57)	329(9.18)	702(19.59)	1881(52.48)	580(16.18)	3.71 ± 0.93
12. I am worried that the HPV vaccine is not administered properly.	102(2.85)	458(12.78)	797(22.24)	1758(49.05)	469(13.09)	3.57 ± 0.97
***Safety***						10.78 ± 2.44

Note. Items assessing the necessity and safety dimensions of the vaccine hesitancy scale were reverse-coded. Consequently, higher scores on these dimensions reflect lower perceived necessity and safety (i.e., greater vaccine hesitancy).

### Health beliefs

In this study, we employed the health belief model to measure students’ health beliefs regarding HPV vaccination. The HBM was validated by Chen et al. [13] (Table 3). This scale includes 13 items across 5 dimensions: perceived benefits of behavior (consisting of 3 items), trust in formal information (consisting of 4 items), perceived disease severity (consisting of 2 items), perceived disease susceptibility (consisting of 3 items), and perceived behavioral barriers (consisting of 1 item). Each item was also scored on a 5-point Likert scale. The overall Cronbach’s α value for the scale was 0.743, indicating acceptable internal consistency.

**Table 3:** Descriptive statistics of HBM.

Term	Strongly disagree N (%)	Disagree N (%)	Neutral/Unsure N (%)	Agree N (%)	Strongly agree N (%)	Score (M ± SD)
1. I think HPV vaccines are important for protecting myself from HPV infection and related diseases.	10(0.28)	17(0.47)	244(6.81)	2183(60.91)	1130(31.53)	4.23 ± 0.61
2. I think the safety of the HPV vaccine can be guaranteed.	15(0.42)	43(1.20)	783(21.85)	1996(55.69)	747(20.84)	3.95 ± 0.72
3. I think the HPV vaccine is effective.	9(0.25)	23(0.64)	458(12.78)	2222(62.00)	872(24.33)	4.10 ± 0.64
***Perceived Benefits of Behavior***						12.28 ± 1.74
4. I trust the HPV vaccine knowledge and services provided by the vaccination clinic and doctor.	11(0.31)	30(0.84)	470(13.11)	2210(61.66)	863(24.08)	4.08 ± 0.65
5. I trust the HPV vaccine knowledge and services provided by CDC, hospitals and other professional institutions.	8(0.22)	23(0.64)	396(11.05)	2254(62.89)	903(25.20)	4.12 ± 0.63
6. I trust HPV vaccine manufacturers and companies.	17(0.47)	101(2.82)	1130(31.53)	1731(48.30)	605(16.88)	3.78 ± 0.77
7. I trust the HPV vaccine information provided by the government.	13(0.36)	26(0.73)	353(9.85)	2239(62.47)	953(26.59)	4.14 ± 0.64
***Trust in Formal Information***						16.13 ± 2.36
8. HPV infection may have serious adverse effects on my health.	64(1.79)	264(7.37)	655(18.28)	1521(42.44)	1080(30.13)	3.92 ± 0.97
9. I think cervical cancer is a serious disease.	24(0.67)	85(2.37)	324(9.04)	1745(48.69)	1406(39.23)	4.23 ± 0.76
***Perceived Disease Severity***						8.15 ± 1.47
10. I don’t think HPV infection can be avoided.	658(18.36)	1777(49.58)	749(20.90)	325(9.07)	75(2.09)	2.27 ± 0.93
11. I think infection by HPV is because of sexual arbitrariness.	402(11.22)	986(27.51)	852(23.77)	1087(30.33)	257(7.17)	2.95 ± 1.15
12. I’m in good health and I’m not afraid of getting HPV.	1283(35.80)	1533(42.77)	445(12.42)	242(6.75)	81(2.26)	1.97 ± 0.98
***Perceived Disease Susceptibility***						7.19 ± 2.36
13. I am very cautious about “getting the HPV vaccine.”	45(1.26)	263(7.34)	772(21.54)	1964(54.80)	540(15.07)	3.75 ± 0.84
***Perceived behavioral barriers***						3.75 ± 0.84

The dimension-level Cronbach’s α values were 0.856 (perceived benefits) and 0.897 (trust in formal information). Lower α values were obtained for perceived disease severity (0.592, 2 items) and perceived susceptibility (0.657, 3 items). Notably, Cronbach’s α is sensitive to the number of items, and values derived from subscales with fewer than three items should be interpreted with caution [14]. The perceived behavioral barriers dimension consisted of a single item and therefore could not yield a Cronbach’s α. Thus, the overall scale Cronbach’s α of 0.743 provides a more robust estimate of reliability.

### Statistical analysis

Data analysis was performed by using IBM SPSS Statistics for Windows, version 29.0 (IBM Corporation, Armonk, NY, USA, 2022; official website: https://www.ibm.com/spss). Univariate analyses were conducted to assess the associations among the variables (Sociodemographics and various dimensions of vaccine hesitancy and the HBM) and HPV vaccination intentions among college students. Variables with *p* < 0.05 in the univariate analyses were entered into a binary logistic regression model to identify significant independent associations with HPV vaccination intentions. Adjusted odds ratios (ORs) with 95% confidence intervals (CIs) were computed, with statistical significance set at *p* < 0.05.

## Results

### Sociodemographic characteristics

Among the study participants, 55.69% (n = 1,996) were female college students, and 44.31% (n = 1,588) were male, with a mean age of 20.63 ± 1.74 years. Regarding main economic source, the majority (95.98%) relied on parental support, while 32.31% reported income from part-time jobs while they were studying. Most participants (78.85%) reported monthly expenditures between 1,000 and 2,000 CNY, and 74.86% reported a monthly family income exceeding 4,000 CNY. The most prevalent parental occupations were employees in enterprises, commercial, and service industries (including self-employed individuals) (27.93%) or workers (27.04%), with 8.23% of the participants having at least one parent in education or medical professions.

Regarding sources of HPV-related information, the five most frequently reported channels were schools/teachers/classmates, social media (WeChat, Weibo, QQ, etc.), the internet (website, email, newsletter, etc.), and hospital/doctor and traditional media (TV/radio/newspapers/magazines).

Notably, nearly half of the respondents had encountered negative information regarding the HPV vaccine. Among the unvaccinated cohort, a substantial proportion (73.16%, n = 2,622) expressed a positive intention to receive the HPV vaccine (Table 1).

### Vaccine hesitancy

Vaccine hesitancy analysis revealed distinct characteristics across dimensions: the necessity dimension had a mean score of 7.56 ± 3.18, which was below the critical threshold of 12. The importance dimension had significantly higher scores (20.56 ± 3.28), exceeding the critical threshold of 15. Notably, the safety dimension had a mean score of 10.78 ± 2.44, which was higher than the critical threshold of 9 (Table 2).

### Health beliefs

The scores of the perceived benefits of behavior, trust in formal information, perceived disease severity, perceived disease susceptibility, and perceived behavioral barriers for the HBM dimensions were 12.28 ± 1.74, 16.13 ± 2.36, 8.15 ± 1.47, 7.19 ± 2.36, and 3.75 ± 0.84, respectively (Table 3).

### Univariate analyses

Univariate analysis revealed significant associations between the intention to receive HPV vaccination and key variables, as detailed in Table 4.

**Table 4:** Univariate associations with the HPV vaccination intention.

Variable	Term	Total, N (%)	HPV Vaccination Intention		*p*-value
			No (n = 962)	Yes (n = 2622)	
Gender	Female	1996(55.69)	302(31.39)	1694(64.61)	**0.000** ^a**^
	Male	1588(44.31)	660(68.61)	928(35.39)	
Age (M ± SD)		20.63 ± 1.74	20.79 ± 1.76	20.58 ± 1.73	**0.001** ^**b**^ **
Average Monthly Consumption Level (CNY)	1000-1500	1839(51.31)	489(50.83)	1350(51.49)	0.533^b^
	1501-2000	987(27.54)	251(26.09)	736(28.07)	
	2001-3000	326(9.10)	89(9.25)	237(9.04)	
	>3000	110(3.07)	35(3.64)	75(2.86)	
Average Monthly Household Income ((CNY)	<1500	96(2.68)	26(2.70)	70(2.67)	**0.030** ^**b**^ *
	1500-2500	224(6.25)	55(5.72)	169(6.45)	
	2501-4000	581(16.21)	146(15.18)	435(16.59)	
	4001-8000	1265(35.30)	322(33.47)	943(35.96)	
	>8000	1418(39.56)	413(42.93)	1005(38.33)	
Main Economic Source	Parents	3440(95.98)	918(95.43)	2522(96.19)	0.305^a^
	Other family members, Relatives, Friends	569(15.88)	142(14.76)	427(16.29)	0.268^a^
	Government fundings	182(5.08)	42(4.37)	140(5.34)	0.239^a^
	Scholarships	597(16.66)	153(15.90)	444(16.93)	0.464^a^
	Part-time job while studying	1158(32.31)	256(26.61)	902(34.40)	**0.000** ^**a**^ **
	Self-employment	68(1.90)	22(2.29)	46(1.75)	0.300^a^
	Student loans	225(6.28)	54(5.61)	171(6.52)	0.320^a^
	Others	238(6.64)	73(7.59)	165(6.29)	0.168^a^
Parents’ Occupations	Personnel of state organs, Party-mass organizations and public institutions	252(7.03)	70(7.28)	182(6.94)	0.728^a^
	Employees in enterprises, commercial, and service industries (including self-employed)	1001(27.93)	223(23.18)	778(29.67)	**0.000** ^**a**^ **
	Workers	969(27.04)	269(27.96)	700(26.70)	0.450^a^
	Farmers	645(18.00)	187(19.44)	458(17.47)	0.173^a^
	Teachers and medical professionals	295(8.23)	99(10.29)	196(7.48)	**0.007** ^**a**^ *
	Others (e.g., military personnel, freelancers)	1204(33.59)	310(32.22)	894(34.10)	0.293^a^
	Unemployed	241(6.72)	72(7.48)	169(6.45)	0.271^a^
Sources/channels of information about HPV	Schools/teachers/classmates	2920(84.15)	747(82.09)	2173(84.88)	**0.047** ^**a**^ *
	Hospital/Doctor	1885(54.32)	469(51.54)	1416(55.31)	**0.050** ^**a**^ *
	Family/relatives/friends	1168(33.66)	286(31.43)	882(34.45)	0.097^a^
	Social media (WeChat, Weibo, QQ, etc.)	2702(77.87)	663(72.86)	2039(79.65)	**0.000** ^**a**^ **
	Internet (website, email, newsletter, etc.)	2620(75.50)	667(73.30)	1953(76.29)	0.071^a^
	TV/radio/newspapers/magazines	1327(38.24)	319(35.05)	1008(39.38)	**0.021** ^**a**^ *
	Advertising	797(22.97)	204(22.42)	593(23.16)	0.646^a^
	Government	705(20.32)	172(18.90)	533(20.82)	0.216^a^
	Community Activities	727(20.95)	178(19.56)	549(21.45)	0.230^a^
	Others	281(8.10)	81(8.90)	200(7.81)	0.301^a^
Heard the negative news about the HPV vaccine?	No	1811(50.53)	517(53.74)	1294(49.35)	**0.020** ^**a**^ *
	Yes	1773(49.47)	445(46.26)	1328(50.65)	
**Vaccine Hesitancy** (M ± SD)	***Necessity***	7.54 ± 3.18	8.68 ± 3.22	7.11 ± 3.06	**0.000** ^**b**^ **
	***Importance***	20.56 ± 3.28	19.54 ± 3.39	20.93 ± 3.16	**0.000** ^**b**^ **
	***Safety***	10.78 ± 2.44	10.82 ± 2.43	10.76 ± 2.44	0.401^b^
**HBM** (M ± SD)	***Perceived Benefits of Behavior***	12.28 ± 1.74	11.68 ± 1.78	12.50 ± 1.67	**0.000** ^**b**^ **
	***Trust in Formal Information***	16.13 ± 2.36	15.51 ± 2.52	16.36 ± 2.25	**0.000** ^**b**^ **
	***Perceived Disease Severity***	8.15 ± 1.47	7.95 ± 1.40	8.23 ± 1.49	**0.000** ^**b**^ **
	***Perceived Disease Susceptibility***	7.19 ± 2.36	7.59 ± 2.35	7.04 ± 2.35	**0.000** ^**b**^ **
	***Perceived behavioral barriers***	3.75 ± 0.84	3.76 ± 0.78	3.75 ± 0.87	0.466^b^

Note. Items assessing the necessity and safety dimensions of the vaccine hesitancy scale were reverse-coded. Consequently, higher scores on these dimensions reflect lower perceived necessity and safety (i.e., greater vaccine hesitancy).

Boldface indicates statistical significance (*p* < 0.05). All the statistical tests were 2-sided. ^a^ *p* value from the Pearson chi-square test.

b *P* value from the Mann‒Whitney test. * *p* < 0.05, ** *p* < 0.01.

Gender (*p*  <  0.001), age (*p*  =  0.001), monthly household income (*p*  =  0.03), main economic source (part-time job while studying, *p*  <  0.001), parents’ occupations (employees in enterprises, commercial, and service industries (including self-employed), *p*  <  0.001), parents’ occupations (teachers and medical professionals, *p*  =  0.007), sources/channels of information about HPV (schools/teachers/classmates, *p*  = 0.047; hospital/doctor, *p*  = 0.05; social media (WeChat, Weibo, QQ, etc.), *p*  <  0.001; TV/radio/newspapers/magazines, *p*  =  0.021) and hearing negative news about the HPV vaccine? (*p*  <  0.02) demonstrated particularly significant correlations.

Notably, the necessity and importance dimensions of HPV vaccine hesitancy were significantly correlated with HPV vaccination intention (all *p* < 0.001).

Furthermore, with the exception of the perceived behavioral barrier dimension (*p*  =  0.466), the perceived benefits of behavior, trust in formal information, perceived disease severity, and perceived disease susceptibility dimensions of the HBM were significantly correlated with HPV vaccination intention (all *p* < 0.001).

### Factors associated with the intention to receive HPV vaccination

The variables that were significant in the univariate analysis were subsequently included in a binary logistic regression analysis. The results are presented in Table 5.

**Table 5:** Binary Logistic Regression for the HPV Vaccination.

Variable	Term	OR, 95% CI	*p*-value
Gender	Female	Ref	
	Male	0.26 (0.22 ~ 0.31)	0.000**
Age		0.98 (0.94 ~ 1.03)	0.469
Average Monthly Household Income(CNY)	<1500	0.94 (0.87 ~ 1.03)	0.173
	1500-2500		
	2501-4000		
	4001-8000		
	>8000		
Main Economic Source	Part-time job while studying	1.20 (1.00 ~ 1.4)	0.056
Parents’ Occupations	Employees in enterprises, commercial, and service industries (including self-employed)	1.43 (1.18 ~ 1.75)	0.000**
	Teachers and medical professionals	0.92 (0.69 ~ 1.24)	0.601
Sources/channels of information about HPV	Schools/teachers/classmates	0.92 (0.73 ~ 1.16)	0.477
	Hospital/Doctor	1.37 (1.15 ~ 1.64)	0.001*
	Social media (WeChat, Weibo, QQ, etc.)	1.16 (0.95 ~ 1.41)	0.145
	TV/radio/newspapers/magazines	0.90 (0.75 ~ 1.08)	0.242
Heard the negative news about the HPV vaccine?	No	Ref	
	Yes	0.98 (0.83 ~ 1.16)	0.830
**Vaccine Hesitancy**	***Necessity***	0.91 (0.88 ~ 0.94)	0.000**
	***Importance***	1.07 (1.03 ~ 1.10)	0.000**
**HBM**	***Perceived Benefits of Behavior***	1.21 (1.12 ~ 1.32)	0.000**
	***Trust in Formal Information***	0.98 (0.93 ~ 1.04)	0.539
	***Perceived Disease Severity***	0.98 (0.91 ~ 1.04)	0.455
	***Perceived Disease Susceptibility***	1.03 (0.99 ~ 1.07)	0.212

Note. Items assessing the necessity and safety dimensions were reverse-coded; higher scores indicate lower perceived necessity/safety (i.e., greater hesitancy). Accordingly, OR < 1 for these dimensions indicates that greater perceived necessity/safety (i.e., lower scores) is associated with higher HPV vaccination intention. OR > 1 for the importance dimension (positively scored) indicates that higher perceived importance is associated with higher vaccination intention.

Boldface indicates statistical significance (*p* < 0.05). All the statistical tests were 2-sided.

* *p* < 0.05, ** *p* < 0.01. Multicollinearity was assessed using variance inflation factors (VIF); all values were below 5, indicating no serious concern.

Hosmer-Lemeshow goodness-of-fit test: χ^2^(8) = 8.232, *p* = 0.411. Nagelkerke R^2^= 0.211.

The results indicated that gender was significantly associated with HPV vaccination intention (OR: 0.26; 95% CI: 0.22 ~ 0.31; *p*  <  0.001). Specifically, compared with female students, male students were more likely to report lower levels of intention.

In addition, having parents employed in enterprises, commercial, or service industries (including self-employment) was positively associated with higher HPV vaccination intention (OR: 1.43; 95% CI: 1.18 ~ 1.75; *p*  <  0.001).

Regarding information sources, obtaining HPV-related information from hospitals or doctors was also a significant positive correlate of vaccination intention (OR: 1.37; 95% CI: 1.15 ~ 1.64; *p*  =  0.001).

Moreover, for HPV vaccine hesitancy, lower perceived necessity (i.e., higher reverse-scored disagreement on vaccine necessity; OR: 0.91; 95% CI: 0.88 ~ 0.94; *p*  <  0.001) and higher perceived importance (OR: 1.07; 95% CI: 1.03 ~ 1.10; *p*  <  0.001) were significantly associated with greater HPV vaccination intention (Table 5).

Furthermore, for the HBM, the perceived benefits of behavior had a significant positive link to HPV vaccination (OR: 1.21; 95% CI: 1.12 ~ 1.32; *p* <  0.001). The other dimensions of the HBM were not significantly associated with the intention to receive the HPV vaccine.

## Discussion

The findings of this study revealed that Chinese medical college students reported a 73.16% overall willingness to receive the HPV vaccine. However, willingness among males was significantly lower than that among females. This gender gap aligns with existing research [15–17]. Its persistence in a medical student sample underscores that professional knowledge does not automatically override sociocultural biases. The pronounced gender disparity primarily arises from the enduring gender-specific labeling of the HPV vaccine as a “cervical cancer vaccine”, which engenders cognitive bias among males, leading them to perceive the vaccine as “gender irrelevant”, even when they are aware of HPV-related male cancers. Consequently, their motivation remains largely altruistic, which may be insufficient to sustain personal vaccination behavior [18]. In reality, males are equally susceptible to health threats from HPV-related malignancies such as oropharyngeal cancer, penile cancer, and anal cancer. To narrow this gap, national immunization programs should transition to gender-neutral HPV vaccination [19], while medical universities could immediately integrate male-specific HPV education into mandatory curricula and offer male-inclusive campus vaccination services—strategies shown to improve vaccination intention [20,21]. While these recommendations are supported by the present findings, the evidence is derived from a medical university sample, and their applicability to the broader college population remains to be examined.

This study revealed that parental employment in enterprise, commerce, or service sectors (inclusive of self-employment) was significantly and positively associated with higher willingness to receive HPV vaccination. Beyond income stability and social security coverage, positions within these sectors are generally linked to more formalized employment than agricultural or informal work [22,23]. Such employment may also provide greater exposure to health information and preventive norms, which, in turn, shape family vaccination decisions. These findings imply that parental occupation, as a social structural variable, may significantly influence family vaccination decisions through combined economic and informational pathways [24]. Therefore, university-based vaccination programs should consider collecting data on parental occupation to identify students with potentially lower vaccination intention and offering them targeted subsidies [25] or parent-centred educational materials [26,27]. Analogous targeted strategies have been recommended in recent HPV vaccination research among parents [28]. From the vantage point of social occupational structure, this research underscores a notable association between parental occupation and medical college students’ willingness to receive HPV vaccination, highlighting the necessity of considering the impact of parental occupation on these students’ vaccine decision-making in efforts to promote HPV vaccine uptake in similar university settings.

Our findings also revealed that the acquisition of HPV-related information from hospitals and doctors was significantly associated with the higher willingness, consistent with prior studies and highlighting the critical role of professional health communication in vaccine decision-making processes [29,30]. Recommendations from hospitals and doctors not only enhance the accuracy of HPV knowledge, alleviate safety concerns, and heighten awareness of vaccination benefits but also foster trust and credibility, which are paramount for the development of positive attitudes and intentions toward vaccination among young people [31]. Research has demonstrated that doctors’ recommendations constitute a key determinant of vaccination uptake [32,33], even among vaccine-hesitant parents [34]. For medical students specifically, this finding suggests an actionable pathway: medical universities could integrate structured, in-person HPV vaccine counseling sessions delivered by clinicians from university-affiliated hospitals into existing preventive medicine clerkships, thereby embedding a trusted provider recommendation directly into the training curriculum [35–37]. Therefore, it is imperative to reinforce the role of doctors as pivotal advocates and to systematically mobilize the accessible hospital resources within medical universities in order to increase HPV vaccination rates among medical students.

Furthermore, in terms of vaccine hesitancy, this study revealed that college students’ perceptions of the necessity of the HPV vaccine significantly and positively correlate with their willingness to receive vaccination; this finding is in line with prior research findings [10,38]. Within the context of the vaccine hesitancy scale, perceived necessity embodies individuals’ subjective evaluation of the irreplaceability of preventive measures. According to the Necessity-Concerns Framework [39], individuals are more likely to accept a medical intervention when perceived necessity outweighs concerns about potential adverse effects. When students fully understand the prevalence of HPV infection and its causal relationship with cancer, the vaccine is perceived as an indispensable “necessity” for safeguarding their health. This psychological impetus effectively counterbalances minor apprehensions regarding side effects associated with the vaccine. In this study, individuals who declined vaccination because of their confidence in robust immunity, good health, or the absence of government mandates demonstrated an inadequate understanding of the necessity of the HPV vaccine. Studies have indicated that skepticism regarding the benefits and necessity of vaccines constitute the primary reason for nonvaccination [40]. Therefore, immunization program interventions in universities should move beyond generic safety reassurance and explicitly juxtapose the necessity of HPV vaccination against safety data—for instance, through structured classroom sessions where students quantitatively compare their personal lifetime risk of HPV infection with the low incidence of serious adverse events [10,41]—thereby enabling perceived necessity to outweigh safety concerns and reduce vaccine hesitancy in this population.

Our findings revealed that the perceived importance of the HPV vaccine was significantly and positively associated with vaccination intention, consistent with previous studies [42]. These findings indicate that when students understand the crucial role of the HPV vaccine in preventing HPV infection and related cancers while improving their own health and that of their families, their willingness to proactively get vaccinated significantly increases. Domestic studies confirm that students who perceive vaccination as less important are less inclined to be vaccinated [43], and international research links perceived importance to vaccination behavior [44,45]. Therefore, rather than solely raising general awareness, medical colleges should embed HPV vaccination importance messaging into existing clinical training (e.g., preventive medicine clerkships), using peer testimonials and case-based discussions to make the vaccine’s value tangible for future healthcare professionals, thereby more effectively enhancing their willingness to be vaccinated.

Finally, this study revealed that the perceived behavioral benefits related to health beliefs have a significant positive link to college students’ willingness to receive the HPV vaccine. Within the HBM framework, perceived benefits represent an individual’s evaluation of the efficacy of a recommended action [46]. When medical students clearly perceive the vaccine’s preventive value, this evaluation directly motivates their vaccination decisions. These findings suggest that individuals’ perceptions of the preventive efficacy and health benefits of the HPV vaccine constitute crucial psychological factors in promoting vaccination decisions, which align with related research on the health belief model [47,48]. Therefore, rather than merely emphasizing protective value in broad terms, future interventions should highlight the practical benefits most relevant to medical students’ dual role as both potential vaccine recipients and future recommenders—for example, personal protection during clinical rotations and their future credibility when counseling patients—thereby optimizing vaccination promotion strategies more effectively [40,49].

## Limitations

This study has several limitations. First, as a cross-sectional survey, it cannot establish causal relationships between variables. HPV vaccination intention was assessed using a single binary (yes/no) item, which may oversimplify this nuanced construct and fails to capture the well-documented intention–behavior gap reported in the vaccination literature; therefore, the reported intention should not be equated with actual vaccine uptake. Second, although known confounding factors were controlled through multivariable analysis, unmeasured confounding variables may still exist. Additionally, the univariate pre-screening approach (*p* < 0.05) used for variable selection, while common, may risk omitting potential confounders or suppressor variables. Third, although the sample size in this study was large, the sample was obtained from a single medical university, and the generalizability of the findings to students in other settings may be limited. The sample was recruited via convenience sampling on WeChat. Medical students likely possess higher health literacy and greater familiarity with HPV than the general college student population, further restricting the external validity of the findings. Additionally, non-response bias cannot be ruled out: students with a greater interest in HPV vaccination may have been more inclined to participate, potentially overestimating the observed willingness. Moreover, the representativeness of the 5,384 respondents relative to the broader student body of the university (e.g., in terms of sex, grade, and major distribution) was not formally assessed, which limits the within-institution generalizability of the results. Fourth, the analytical strategy adopted in this study treated health beliefs and vaccine hesitancy variables as parallel predictors in regression models, consistent with examining their independent associations. No mediation, moderation, or structural equation modeling was performed to formally test the integrated structure of these two theoretical frameworks. Consequently, the findings cannot determine how the constructs from the HBM and vaccine hesitancy models causally relate to or interact with each other in shaping vaccination intention. Furthermore, two HBM dimensions—perceived disease severity and perceived disease susceptibility—showed relatively low internal consistency (Cronbach’s α = 0.592 and 0.657, respectively), which is partially attributable to their small number of items (2 and 3 items, respectively), consistent with the known sensitivity of Cronbach’s α to item count [14]. This level of reliability may have attenuated observed effect sizes and reduced statistical power to detect associations with HPV vaccination intention in the logistic regression models. Therefore, the non-significant findings for these two dimensions should be interpreted with caution, as they do not definitively rule out the potential theoretical and practical relevance of these constructs to HPV vaccination intention. Future studies could improve the measurement of these constructs by increasing the number of items in these subscales to enhance internal consistency and statistical power. Future research employing longitudinal or experimental designs and advanced path-analytic techniques is needed to directly test hypothesized integrative pathways. Moreover, such designs should be used not only to test hypothesized integrative pathways but also to evaluate interventions that can improve the current insufficient HPV vaccination coverage. Fifth, the final logistic regression model explained approximately 21% of the variance in HPV vaccination intention (Nagelkerke R^2^ = 0.211), indicating modest explanatory power and that a substantial portion of variance remains unexplained. Additional constructs not captured by the current instruments—such as HPV-specific knowledge, peer norms, perceived access or cost barriers, and prior sexual health education,which are particularly relevant to student populations—may account for this remaining variance. Future studies aiming to build more comprehensive predictive models should consider incorporating these variables to improve model explanatory power and identify more robust determinants of HPV vaccine uptake.

## Conclusions

In this study, the intentions to receive HPV vaccination among unvaccinated college students at Guangdong Medical University were assessed on the basis of vaccine hesitancy and the health belief theory. This study revealed that college students exhibit a high willingness to receive the HPV vaccine. Several positive factors associated with the willingness to receive vaccination were identified, including female gender, parents’ occupations (employees in enterprises, commercial, and service industries (including self-employed)), and information sources (hospitals or doctors). Furthermore, the study indicated that perceptions of the necessity, importance, and benefits of vaccination positively linked to the willingness to receive vaccination.

The study findings emphasize that, to increase Chinese medical college students’ willingness to receive HPV vaccination, targeted interventions are recommended: integrating structured clinician-delivered counseling into preventive medicine clerkships, collecting parental occupation data to guide targeted subsidies and education, and embedding classroom sessions that compare personal HPV infection risk with vaccine safety data. Furthermore, government evaluation of gender-neutral vaccination policies and the implementation of male-inclusive campus vaccination services are urged, although the generalizability of these findings to non-medical college populations requires further investigation.

## Supporting information

S1 File Raw data (N = 3584). (XLSX)

## References

[1] Bruni L, Albero G, Serrano B, Mena M, Collado J, Gómez D. boo. ICO/IARC Information Centre on HPV and Cancer (HPV Information Centre). 2023.

[2] Gao D, Zhao G, Wang X, Juan J, Shi Y, Xu T, et al. Association between high-risk human papillomavirus infection and cervical cytology in health check-up women - 23 PLADs, China, 2023. China CDC Wkly. 2025;7:327–33. 40225780 10.46234/ccdcw2025.053 PMC11982919

[3] Shi Y, Liu R, Yu H, Fu Z, Guo W. Sexual debut among college students in China: effects of family context. J Biosoc Sci. 2022;54(6):1004–23. doi: 10.1017/S0021932021000523 34730078

[4] Chen J, Zhang Z, Pan W, Song Y, Zheng L, Li L, et al. Estimated human papillomavirus vaccine coverage among females 9-45 years of age - China, 2017-2022. China CDC Wkly. 2024;6:413–7. 38854753 10.46234/ccdcw2024.080 PMC11153868

[5] Li W, Jin Y, Li X, Jin M. Human papillomavirus and vaccine knowledge, willingness, and uptake among university students: a systematic review and meta-analysis. BMC Public Health. 2025;25(1):4268. doi: 10.1186/s12889-025-25626-4 41419843 PMC12717715

[6] Rosenstock IM, Strecher VJ, Becker MH. Social learning theory and the Health Belief Model. Health Educ Q. 1988;15(2):175–83. doi: 10.1177/109019818801500203 3378902

[7] Zheng D, Lekdamrongkul P, Gao X, Sriyuktasuth A. Exploring theoretical models and frameworks used to explain factors influencing breast cancer screening participation: a scoping review. Int J Womens Health. 2025;17:5639–56. doi: 10.2147/IJWH.S553089 41480293 PMC12753854

[8] Qayum I. Top ten global health threats for 2019: the WHO list. J Rehman Med Inst. 2019;5:1–2.

[9] WHO. Report of the SAGE working group on vaccine hesitancy. Geneva: World Health Organization; 2014.

[10] Chen C, Chen T, Huang M, Huang Y, Zhang L, Li P. Factors associated with HPV vaccine hesitancy among college students: a cross-sectional survey based on 3Cs and structural equation model in China. Hum Vaccin Immunother. 2024;20(1):2309731. doi: 10.1080/21645515.2024.2309731 38314749 PMC10854271

[11] Wei Z, Liu Y, Zhang L, Sun X, Jiang Q, Li Z, et al. Stages of HPV vaccine hesitancy among guardians of female secondary school students in China. J Adolesc Health. 2023;72(1):73–9. doi: 10.1016/j.jadohealth.2022.08.027 36229401 PMC9746349

[12] HPV vaccine for adolescents in China: what is the next step?. Lancet Reg Health West Pac. 2026;66:101810. 41647887 10.1016/j.lanwpc.2026.101810 PMC12869286

[13] Chen L, Sun X, Luo J, Zhang Y, Ha Y, Xu X, et al. A case-control study on factors of HPV vaccination for mother and daughter in China. Vaccines (Basel). 2023;11(5):976. doi: 10.3390/vaccines11050976 37243080 PMC10224422

[14] Eisinga R, Grotenhuis M te, Pelzer B. The reliability of a two-item scale: Pearson, Cronbach, or Spearman-Brown?. Int J Public Health. 2013;58(4):637–42. doi: 10.1007/s00038-012-0416-3 23089674

[15] Chen G, Wu B, Dai X, Zhang M, Liu Y, Huang H, et al. Gender differences in knowledge and attitude towards HPV and HPV Vaccine among College Students in Wenzhou, China. Vaccines (Basel). 2021;10(1):10. doi: 10.3390/vaccines10010010 35062671 PMC8779512

[16] Dorji T, Nopsopon T, Tamang ST, Pongpirul K. Human papillomavirus vaccination uptake in low-and middle-income countries: a meta-analysis. EClinicalMedicine. 2021;34:100836. doi: 10.1016/j.eclinm.2021.100836 33997733 PMC8102703

[17] Dai Z, Si M, Su X, Wang W, Zhang X, Gu X, et al. Willingness to human papillomavirus (HPV) vaccination and influencing factors among male and female university students in China. J Med Virol. 2022;94(6):2776–86. doi: 10.1002/jmv.27478 34825712 PMC9299831

[18] Shen F, Du Y, Cao K, Chen C, Yang M, Yan R, et al. Acceptance of the human papillomavirus vaccine among general men and men with a same-sex orientation and its influencing factors: a systematic review and meta-analysis. Vaccines (Basel). 2023;12(1):16. doi: 10.3390/vaccines12010016 38250829 PMC10819436

[19] Dykens JA, Peterson CE, Holt HK, Harper DM. Gender neutral HPV vaccination programs: Reconsidering policies to expand cancer prevention globally. Front Public Health. 2023;11:1067299. doi: 10.3389/fpubh.2023.1067299 36895694 PMC9989021

[20] WHO. Human papillomavirus vaccines: WHO position paper (2022 update). Wkly Epidemiol Rec. 2022;97:645–72.

[21] Xie B, Xiao L, Peng L. Towards HPV elimination: closing the gender gap by vaccinating and screening men. Lancet Reg Health Am. 2025;50:101251. doi: 10.1016/j.lana.2025.101251 41035548 PMC12481096

[22] Xue J, Gao W, Guo L. Informal employment and its effect on the income distribution in urban China. China Economic Review. 2014;31:84–93. doi: 10.1016/j.chieco.2014.07.012

[23] Lund F. Work‐related social protection for informal workers. Int Social Security Review. 2012;65(4):9–30. doi: 10.1111/j.1468-246x.2012.01445.x

[24] Rivera AF, Dussault JM, Oudin Doglioni D, Chyderiotis S, Sicsic J, Barret A-S, et al. Sociodemographic determinants of HPV vaccine awareness, uptake, and intention among parents of adolescents in France 2021-22. Hum Vaccin Immunother. 2024;20(1):2381300. doi: 10.1080/21645515.2024.2381300 39105306 PMC11305024

[25] Xiang J, Sun X. Socioeconomic disparities in HPV vaccine uptake: multivariable analysis of vaccination data from Tianjin (2018–2023). Frontiers in Public Health. 2025;13:1428267. 40084203 10.3389/fpubh.2025.1428267 PMC11903397

[26] Ragan KR, Bednarczyk RA, Butler SM, Omer SB. Missed opportunities for catch-up human papillomavirus vaccination among university undergraduates: Identifying health decision-making behaviors and uptake barriers. Vaccine. 2018;36(2):331–41. doi: 10.1016/j.vaccine.2017.07.041 28755837

[27] Vu M, Bednarczyk RA, Escoffery C, Getachew B, Berg CJ. Human papillomavirus vaccination among diverse college students in the state of Georgia: who receives recommendation, who initiates and what are the reasons?. Health Educ Res. 2019;34(4):415–34. doi: 10.1093/her/cyz014 31081024 PMC6646951

[28] Rivera AF, Dussault JM, Oudin Doglioni D, Chyderiotis S, Sicsic J, Barret A-S, et al. Sociodemographic determinants of HPV vaccine awareness, uptake, and intention among parents of adolescents in France 2021-22. Hum Vaccin Immunother. 2024;20(1):2381300. doi: 10.1080/21645515.2024.2381300 39105306 PMC11305024

[29] Okoli GN, Soos AE, Etsell K, Grossman Moon A, Kimmel Supron H, Grewal A, et al. Socioeconomic and health-related characteristics associated with initiation and completion of human papillomavirus vaccination among males in the United States: an in-depth systematic review and meta-analysis. Behav Med. 2025;51(3):185–206. doi: 10.1080/08964289.2024.2447358 39851094

[30] Constable C, Ferguson K, Nicholson J, Quinn GP. Clinician communication strategies associated with increased uptake of the human papillomavirus (HPV) vaccine: a systematic review. CA Cancer J Clin. 2022;72(6):561–9. doi: 10.3322/caac.21753 35969145

[31] Btoush R, Kohler RK, Carmody DP, Hudson SV, Tsui J. Factors that influence healthcare provider recommendation of HPV vaccination. Am J Health Promot. 2022;36(7):1152–61. doi: 10.1177/08901171221091438 35442819 PMC10326631

[32] Liu A, Ho FK, Chan LK, Ng JY, Li SL, Chan GC, et al. Chinese medical students’ knowledge, attitude and practice towards human papillomavirus vaccination and their intention to recommend the vaccine. J Paediatr Child Health. 2018;54(3):302–10. doi: 10.1111/jpc.13693 28876498

[33] Alghanmi R, Alkhalawi E, Albeladi R, Albeladi S, Alghamdi M, Fayoumi A, et al. Parental knowledge and acceptance of HPV vaccine in Rabigh’s School, Saudi Arabia. Ann Glob Health. 2026;92(1):13. doi: 10.5334/aogh.4866 41659211 PMC12880011

[34] Willis DE, Moore R, Selig JP, Shafeek Amin N, Li J, Watson D, et al. Pediatric HPV vaccination: provider recommendations matter among hesitant parents. Vaccine. 2024;42(25):126166. doi: 10.1016/j.vaccine.2024.126166 39079809 PMC11401755

[35] Yang H, Thongjit S, Yangyuen S. The effects of a protection motivation theory-based online educational program on HPV vaccination intention among female medical students in Hubei province, China: a randomized controlled trial. J Educ Health Promot. 2025;14:225. doi: 10.4103/jehp.jehp_1530_24 40575532 PMC12200204

[36] Sutton S, Azar SS, Evans LK, Murtagh A, McCarthy C, John MS. HPV knowledge retention and concurrent increase in vaccination rates 1.5 years after a novel hpv workshop in medical school. J Cancer Educ. 2023;38(1):240–7. doi: 10.1007/s13187-021-02106-y 34669178

[37] Schnaith AM, Evans EM, Vogt C, Tinsay AM, Schmidt TE, Tessier KM, et al. An innovative medical school curriculum to address human papillomavirus vaccine hesitancy. Vaccine. 2018;36(26):3830–5. doi: 10.1016/j.vaccine.2018.05.014 29778518 PMC6819154

[38] Hu SL, Yu Y, Zhang WY, Zhang YJ, Li RP, Xu AQ, et al. Knowledge, attitude and practices of adult vaccine among the staff of vaccination units in Shandong Province. Zhonghua Yu Fang Yi Xue Za Zhi. 2024;58(8):1252–5. doi: 10.3760/cma.j.cn112150-20240305-00188 39142897

[39] Foot H, La Caze A, Gujral G, Cottrell N. The necessity-concerns framework predicts adherence to medication in multiple illness conditions: a meta-analysis. Patient Educ Couns. 2016;99(5):706–17. doi: 10.1016/j.pec.2015.11.004 26613666

[40] El-Hadad S, Sachs MK, Barrense-Dias Y, Granell JCS, Niggli A, Leeners B. HPV vaccination among young adults in Switzerland: a cross-sectional study. BMJ Open. 2025;15(2):e089681. doi: 10.1136/bmjopen-2024-089681 39938962 PMC11831261

[41] Wang X, Yan B, Wang X. Effects of message framing on HPV Vaccination decision-making: a survey experiment among Chinese male college students. Hum Vaccin Immunother. 2026;22(1):2606516. doi: 10.1080/21645515.2025.2606516 41480936 PMC12773642

[42] You D, Han L, Li L, Hu J, Zimet GD, Alias H, et al. Human Papillomavirus (HPV) vaccine uptake and the willingness to receive the hpv vaccination among female college students in China: a multicenter study. Vaccines (Basel). 2020;8(1):31. doi: 10.3390/vaccines8010031 31963370 PMC7157221

[43] Liu L, Lin S, Gu C. Human papillomavirus (HPV) vaccination intentions and associated factors among female Chinese undergraduate students. Asia-Pacific Journal of Oncology Nursing. 2024;11:100458. doi: 10.1016/j.apjon.2024.100458

[44] Brumbaugh KQ, de Figueiredo A, Gellert FR, Casas FR, McCoy T, Larson HJ, et al. Drivers of HPV vaccine hesitancy in New York and Florida. Vaccine. 2025;61:127395. doi: 10.1016/j.vaccine.2025.127395 40540784

[45] Maletin N, Denda N, Ljubičić A, Velicki R, Patić A, Golušin Z, et al. Knowledge and attitudes regarding human papillomavirus vaccination among future healthcare workers in Serbia. Vaccines (Basel). 2024;13(1):11. doi: 10.3390/vaccines13010011 39852791 PMC11768562

[46] Janz NK, Becker MH. The Health Belief Model: a decade later. Health Educ Q. 1984;11(1):1–47. doi: 10.1177/109019818401100101 6392204

[47] Song S-Y, Guo Y, Li Y-H, Wang Z, Gao W. Analysis of factors influencing HPV vaccination intention among Chinese college students: structural equation modeling based on health belief theory. Front Public Health. 2025;12:1510193. doi: 10.3389/fpubh.2024.1510193 39949340 PMC11821927

[48] Alizadeh L, Keshavarz Z, Shalbaf A, Iranpour S. Iranian women’s perceptions of human papillomavirus and barriers to vaccination: a qualitative study based on the health belief model. BMC Public Health. 2025;26(1):5. doi: 10.1186/s12889-025-25583-y 41286815 PMC12764155

[49] Wang W. The role of personal health beliefs and altruistic beliefs in young Chinese adult men’s acceptance of the human papillomavirus vaccine. Sci Rep. 2024;14(1):20341. doi: 10.1038/s41598-024-71494-0 39242754 PMC11379873

